# Diagnosis and Management of Pulmonary Manifestations of Telomere Biology Disorders

**DOI:** 10.1007/s11899-023-00720-9

**Published:** 2023-12-30

**Authors:** Kathryn T. del Valle, Eva M. Carmona

**Affiliations:** Division of Pulmonary and Critical Care Medicine, Department of Medicine, Mayo Clinic, Rochester, MN USA

**Keywords:** Lung fibrosis, Telomere biology disorders, Telomere length, Interstitial lung disease

## Abstract

**Purpose of Review:**

Telomere biology disorders (TBD) are a group of genetic disorders characterized by premature shortening of telomeres, resulting in accelerated aging of somatic cells. This often leads to major multisystem organ dysfunction, and TBDs have become increasingly recognized as a significant contributor to numerous disease processes within the past 10–15 years. Both research and clinical practice in this field are rapidly evolving.

**Recent Findings:**

A subset of patients with TBD suffers from interstitial lung disease, most commonly pulmonary fibrosis. Often, the clinical presentation is indistinguishable from other forms of lung fibrosis. There are no pathognomonic radiographic or histological features, and a high level of suspicion is therefore required. Telomere evaluation is thus crucial to establishing the diagnosis.

**Summary:**

This review details the clinical presentation, objective evaluation, indicated genetic testing, and recommended management strategies for patients affected by interstitial lung disease associated with TBDs. Our goal is to empower pulmonologists and other healthcare professionals who care for these patients to provide appropriate and personalized care for this population.

## Introduction

Telomere biology disorders (TBDs) are a heterogeneous group of genetic conditions characterized by accelerated aging of somatic cells due to prematurely shortened telomere lengths, frequently leading to organ dysfunction [1•, 2]. They are also often referred to clinically as “short telomere syndromes” (STS). Telomeres are the protective caps at the ends of chromosomes that prevent DNA damage and maintain chromosomal integrity. Patients affected by TBDs may present with hematopoietic, hepatobiliary, skin, and pulmonary manifestations, among others—in other words, it predominantly affects those systems with high lifetime replicative demands on a cellular level [3–5].

Over the past decade, TBDs have become increasingly recognized in the pathophysiology of fibrotic interstitial lung disease (ILD). ILD describes various parenchymal lung disorders involving tissue within and surrounding the alveoli [5]. This heterogeneous group includes entities like idiopathic pulmonary fibrosis (IPF), ILD associated with connective tissue or autoimmune disease, hypersensitivity pneumonitis (HP), interstitial pneumonia with autoimmune features (IPAF), combined pulmonary fibrosis and emphysema (CPFE), and pleural parenchymal fibroelastosis (PPFE). Shortened telomere length, which occurs uniformly with advancing age, has been identified as a biomarker associated with increased mortality among large cohorts with different clinical phenotypes of pulmonary fibrosis [6]. The risk of pathogenic genetic variants leading to prematurely shortened telomeres is magnified and accelerated at a younger age.

It is widely recognized that fibrotic ILD often runs in families [7], commonly referred to as familial pulmonary fibrosis (FPF). The European Respiratory Society (ERS) statement and the Pulmonary Fibrosis Foundation (PFF) genetic testing working group define FPF as any fibrotic ILD affecting at least two first- or second-degree family members (must be blood relatives) [8••, 9••]. Several studies have shown that mutations in telomere-related genes are commonly implicated in these cases [10, 11]. In one study, 37% of patients with familial disease and 25% with sporadic cases had shortened telomeres, defined within that cohort as telomere lengths < 10th percentile [12]. More specifically, the loss of function in the telomerase reverse transcriptase (TERT) accounted for the most significant subset of mutations found in patients with FPF. Other common mutations associated with FPF are *DKC1*, *NAF1*, *PARN*, *RTEL1*, *TINF2*, *TR*, and *ZCCHC8*. Most of the mutations are heterozygous and cause autosomal dominant disease. However, some can be recessive and X-linked, like *DKC1*. Interestingly, the association of pulmonary fibrosis and bone marrow failure within the same family predicts the presence of a germline defect in telomere-related genes with at least 80% specificity [13]. However, most patients with isolated TBD-associated lung disease are clinically indistinguishable and can only be identified by genetic sequencing [10].

This review will summarize the clinical presentation, diagnosis, genetic evaluation, interpretation, and management of TBD-associated lung disease to empower healthcare professionals to take optimal care of this unique group of patients.

## Clinical Presentation

The diagnosis of TBD-associated ILD requires clinical and genetic evaluation, often further supported by radiographic and pathologic findings. Like other patients with parenchymal lung disease, TBD-associated ILD patients may present with non-specific symptoms. Exertional dyspnea, dry cough, and decreased exertional tolerance are often the presenting symptoms. Obtaining a detailed individual and familial medical history is essential. Specifically, patients should be asked about family members’ pulmonary disease history, premature hair graying, unexplained cytopenias or other hematologic abnormalities, liver cirrhosis (particularly cryptogenic), or other hepatic abnormalities or known telomere conditions such as dyskeratosis congenita [1•, 8••]. Most common associated manifestations of TBD are listed in Table 1.

**Table 1:** Common manifestations of TBDs

Hematologic/bone marrow	Pulmonary	Hepatic	Gastrointestinal	Skin/hair	Malignancy
Aplastic anemia	Pulmonary fibrosis*	Nodular regenerative hyperplasia	Enterocolitis	Premature gray hair*	Hematologic cancer (e.g., acute myeloid leukemia)
Dyskeratosis congenita*	Other forms of ILD (chronic HP, NSIP, etc.)	Cryptogenic cirrhosis	Enteropathy	Alopecia	Head and neck cancers (squamous cell)
Macrocytosis*	Emphysema	Portal hypertension	Esophageal strictures	Upper chest/neck reticular pigmentation	Gastrointestinal cancer
Cytopenias* (neutropenia, lymphopenia, thrombocytopenia)	Hepatopulmonary syndrome			Dystrophic nails	Skin cancer (squamous cell)
Acute myeloid leukemia				Mucosal leukoplakia	Anogenital cancers
Myelodysplastic syndrome					

Adapted from [14]

*denotes TBD criteria per ERS [8••]

Physical exam findings are like other forms of fibrotic ILD. They may include inspiratory (“velcro”) crackles on lung auscultation, clubbing, and potentially other findings related to extrapulmonary manifestations of TBDs, such as diffuse pallor if they have anemia or manifestations of hepatic dysfunction (ascites, etc.).

## Evaluation

Initial evaluation of pulmonary manifestations of TBDs should include complete pulmonary function testing (plethysmography and spirometry), which typically demonstrates a restrictive pattern and diffusing lung capacity of carbon monoxide (DLCO), which is commonly reduced depending on the extent/severity of parenchymal involvement. Measurement of oxygen saturation at rest and with exertion and a 6-min walk distance (6MWD) are recommended in most cases. Basic laboratory evaluation, including complete blood count (CBC) with differential and comprehensive metabolic panel (CMP), looking for extrapulmonary manifestations should be included as well. In fact, anemia has been reported in patients with TBD in about 7%, macrocytosis in 24–41%, and thrombocytopenia in 8–54% [15]. Myelodysplasia and acute leukemia in patients with fibrotic ILD should also prompt TBD evaluation. Hepatic involvement can range from asymptomatic elevation of liver enzymes in 5–27% of patients to liver cirrhosis and hepatopulmonary syndrome in more severe cases [16]. The need for additional evaluation should be individualized and based on the presence of other manifestations known to be associated with TBD (see Table 1).

A thorough investigation of alternative etiologies of ILD should also be conducted, including a comprehensive history of possible exposures and medications known to be associated with lung fibrosis, autoimmune studies (antinuclear antibody (ANA), rheumatoid factor (RF), anti-cyclic citrullinated peptide (CCP), antineutrophil cytoplasmic antibodies (ANCA), and serologic screenings for other connective tissue diseases), and antigen testing if there is suspicion for HP. Not uncommonly, patients may present with positive results and no clinical evidence of autoimmune or rheumatological processes. Elevation of antigen panels is also frequently seen in a subset of TBD patients. Careful interpretation of these results in conjunction with genetic evaluation is essential and often requires a multidisciplinary approach (rheumatology, hematology hepatology, genetic counseling, genetic researchers, pulmonology, immunology, radiology, and pathology).

**Fig. 1:**
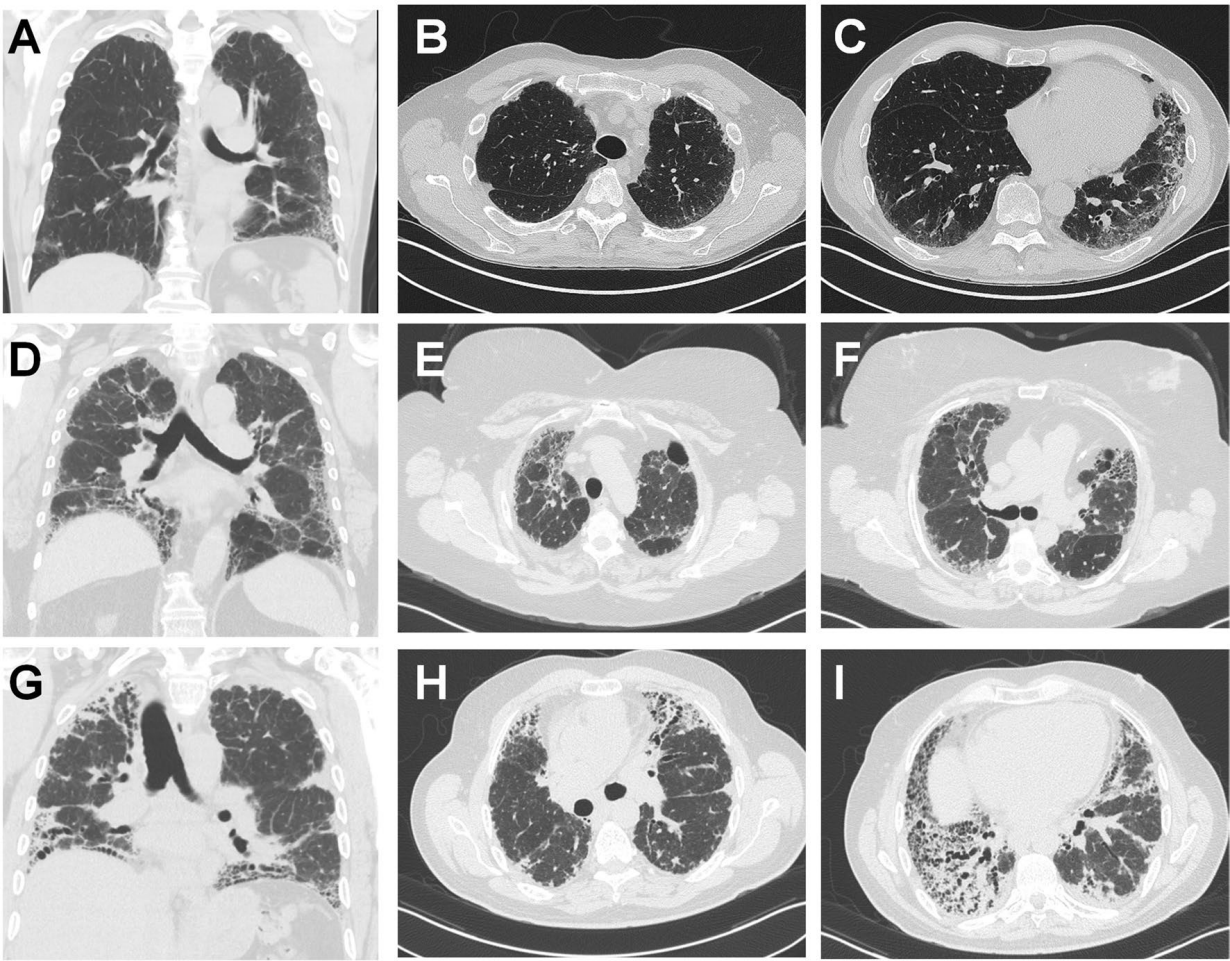
Representative high-resolution computed tomography (HRCT) coronal (**A**, **D**, and **G**) and axial (**B**, **C**, **E**, **F**, **H**, and **I**) images from adults with pulmonary fibrosis in the setting of pathogenic variants in telomere-related genes. **A**–**C** A 76-year-old male heterozygous for RTEL1 (c.3724 + 139 G > A). **D**–**F** A 64-year-old female heterozygous for *TERT (*c.1864 C > T, (p.R622C)). **G**–**I** A 59-year-old male heterozygous for TERT (c.2030 G > A, p.Gly677Asp)

Radiographic evaluation—specifically, high-resolution computed tomography (HRCT) imaging—plays a vital role in diagnosing ILD associated with TBD. It manifests in multiple patterns, as shown in Fig. 1, and poses significant diagnostic challenges. While a clinical diagnosis of IPF is generally associated with usual interstitial pneumonia (UIP) on imaging, cohort studies of patients with fibrotic ILD related to short telomeres demonstrate notable imaging diversity. For instance, in a cohort of 26 patients with telomeres length ≤ 10th percentile and fibrotic ILD, 65% (17/26) showed a typical or probable UIP pattern on HRCT based on Fleischner criteria. In contrast, the remaining 35% were classified as indeterminate or inconsistent with UIP [17]. The same study analyzed pathologic specimens to classify disease patterns further, and 47% (8/17) of the patients with typical or probable UIP on HRCT had morphologic features consistent with UIP; 41% (7/17) were ultimately diagnosed via a multidisciplinary approach with IPF and 29% (5/17) with FPF. Other patients were found to have alternative patterns, such as PPFE, chronic HP, and non-specific interstitial pneumonia (NSIP). The overall cohort revealed a large spectrum of radiologic and pathologic patterns, underscoring the complexity and heterogeneity of how fibrotic lung diseases manifest in this population [17]. Another study by Newton et al. found similar diversity among radiographic patterns within a telomere-related fibrosis cohort. Also, it demonstrated a clear progression of these conditions over time, regardless of specific patterns [16]. Telomere length itself—i.e., the degree of how severely shortened telomeres are—has also been shown to correlate with progression [18]. Examples of HRCT images from patients with ILD in the context of pathogenic variants in telomere-related genes are seen in Fig. 1.

Additionally, we recommend screening for ILD in patients diagnosed with short telomeres or TBD, even without respiratory symptoms (see below), given how commonly these patients develop pulmonary involvement. Among one of the larger published cohorts of TBD patients, 54% (*n* = 135) had fibrotic ILD with various radiological patterns (UIP, HP, PPFE, etc.) (3). Moreover, radiographic findings suggestive of ILD may precede clinically apparent ILD.

Pulmonary hypertension (PH), specifically World Health Organization (WHO) Group 3 PH associated with ILD, may affect patients with TBD-associated ILD. Therefore, we advise consideration of screening echocardiogram to evaluate for elevated right ventricular systolic pressure (RVSP), RV enlargement and dysfunction, or other findings suggestive of PH. Pending findings, referral to a PH specialty team, may be warranted.

Finally, the role of lung biopsy among these patients remains controversial. Generally, lung biopsy would only be clinically useful (and therefore recommended) if an alternative diagnosis is strongly suspected, and the clarification would meaningfully change management. In our experience, this is the case in a small minority of cases.

## Genetic Testing

As previously discussed, TBD-associated lung fibrosis has no pathognomonic radiological or histological characteristics. Therefore, a high level of suspicion is required to ensure that healthcare providers do not overlook the diagnosis. Genetic evaluation is thus crucial to establishing the diagnosis and avoiding invasive procedures such as lung biopsy in the right clinical scenario.

As genetic evaluation is still inconsistently used, the PFF genetic testing working group and the ERS task force have published recommendations that guide healthcare professionals caring for these patients [8••, 9••]. Based on their recommendations, telomere evaluation should be offered to any of the following:

- Patients with pulmonary fibrosis and familial history of fibrosis (one or more first-degree family members with fibrotic ILD)
- Patients with a relative carrying a pathogenic/likely pathogenic variant known to cause fibrotic ILD
- Patients with pulmonary fibrosis and personal or family history of TBD manifestations (Table 1)
- Patients with the onset of fibrotic lung disease before the age of 50 years

For the purpose of this review, we will mainly discuss the genetic evaluation of TBD-associated ILD. However, additional genetic testing, including surfactant protein genes, is recommended if no telomere-related alterations are identified.

If TBD-associated ILD is suspected, genetic sequencing of telomere-related genes and telomere length evaluation are the two main clinical tests available. Telomere length is generally measured in peripheral blood, and while several methods are available, flow-FISH is the preferred clinical test due to its reproducibility [19, 20]. As telomere length decreases with age, flow-FISH data is compared with a control population and adjusted for age. Age-adjusted telomere length is considered short if telomere length is equal to or less than the 10th percentile and very short if telomere length is less than the 1st percentile in lymphocytes and/or granulocytes. Patients presenting with hematological disorders tend to have shorter telomeres than those with mainly lung or liver disease. However, normal telomere length has been described in patients with pathogenic mutations in TBD genes [20, 21]. Therefore, a normal flow-FISH test should not prevent further evaluation if TBD-related ILD is suspected. Potential pitfalls of using flow-FISH testing include interassay variability, measuring only mean telomere length, lack of ability to measure tissue-specific telomere length, and the possibility of missing silent genetic carriers who may have telomere length at the lower end of the normal range (3).

On the contrary, adult patients with shortened telomere lengths have a < 20% positivity rate for a TBD-associated pathogenic variant, suggesting inheritance of the shorter telomeres, epigenetic, and non-genetic mechanisms of telomere length regulation (3). These patients are also at risk of developing lung fibrosis and other TBD manifestations [12, 20, 22].

There are different genetic sequencing modalities. The most common are whole-genome sequencing (WGS), whole-exome sequencing (WES), and targeted gene sequencing panels. Each technique has advantages and disadvantages, such as cost, availability, and coverage. In our institution, and for routine clinical use, we almost exclusively use a targeted gene panel that includes the most commonly described telomere and non-telomeres genes [8••, 9••]. Peripheral blood, saliva, buccal swabs, or skin biopsies are all accepted samples for genomic DNA isolation. Saliva and buccal swab samples are most convenient for “mail-in” samples.

Reporting genetic variants follows the American College of Medical Genetics standardized definitions, including pathogenic, likely pathogenic, uncertain significance, likely benign, and benign [23, 24]. Positive results (pathogenic or likely pathogenic) are easier to interpret and can be found in up to 30% of patients with FPF. Interpreting negative results (uncertain significance, likely benign, and benign), especially those reported as variants of uncertain significance (VUS), can be challenging and require genetic expertise. The presence of a VUS should not be interpreted as benign, and it generally means that there is insufficient data to determine whether the variant is pathogenic. Based on emerging information from additional clinical genetic databases and functional studies, a VUS can be later reclassified as either likely pathogenic, pathogenic, or benign. If available at your institution, working with a genetic research team can be very helpful and provide further insights on otherwise reported negative results. For instance, a negative result from WES or targeted gene sequencing panel must be interpreted cautiously as potentially pathogenic variants present in the intronic areas of the gene can be missed. Additionally, they can evaluate the potentially pathogenic likelihood of a VUS, depending on the type of mutation, location, and other characteristics. Their collaboration is critical to suggest further research studies, including family testing [9••].

## Genetic Counseling

Genetic counseling should be offered to all patients with suspected TBD-related ILD even before genetic testing is ordered. Patients need to clearly understand the different genetic testing options, insurance concerns, and their rights. After results are available, the genetic counselors assist patients and family members regarding inheritance patterns, different expression patterns, and penetrance. They suggest further screening to relatives and recommend additional clinical care.

## Screening for Pulmonary Fibrosis

Due to the high prevalence of lung fibrosis in carriers of TBD-pathogenic variants (15–55%) [21, 25, 26], it is recommended that all symptomatic family members of TBD-confirmed individuals undergo evaluation for ILD. Our institution recommends a complete pulmonary evaluation, including CBC, CMP, complete PFTs, DLCO, HRCT, flow-FISH, and targeted genetic testing, with potential referral to the full TBD clinic if results are suggestive of disease. For the asymptomatic members, we recommend targeted genetic testing and if abnormal obtaining a baseline evaluation with complete CBC, CMP, PFTs, DLCO, and HRCT if PFTs or DLCO are abnormal, otherwise HRCT at 45–50 years or 10–15 years before the first manifestation of the affected family member. These are similar to other available recommendations [27] and consider the known genetic anticipation of telomere shortening. While recommending asymptomatic family members of a relative with likely a pathogenic mutation to be tested, the ERS statement also suggests following the guidance of local health and insurance policies. They additionally advise against testing asymptomatic relatives under the age of 18 until they can make their own informed decision due to the psychological, social, and financial impact that results may have. Several studies are underway to understand better early fibrotic changes, also called interstitial lung abnormalities (ILA), in individuals with family members affected with FPF (clinical tials.gov).

## Management

As with other forms of fibrotic lung disease, managing TBD-associated ILD can be challenging and typically requires a multifaceted approach. Avoiding tobacco and inhaled irritants such as e-cigarettes, vaping, and other recreational drugs is vital to minimize lung injury. Occupational exposures to silica, aluminum, lead, and organic antigens like molds and bird feathers should also be avoided. Medications with known lung toxicity (e.g., amiodarone, nitrofurantoin, checkpoint inhibitors) and radiation exposure should be avoided or closely monitored in this population.

Unlike other forms of ILD, especially those associated with autoimmune disorders, steroids and steroid-sparing immunosuppressant agents (mycophenolate, azathioprine) should generally not be used in patients with TBD. In an analysis of patients with IPF and short telomeres, treatment with prednisone and additional immunosuppressants was associated with worse survival, more notably than those without TBD [28]. Similarly, patients with chronic HP treated with mycophenolate have been shown to have worse outcomes [29]. Like IPF and other forms of ILD, the role of steroids in ILD exacerbations among the TBD cohort remains unclear, with data quite mixed at this point.

The role of antifibrotics (pirfenidone and nintedanib) in TBD-related ILD is an evolving area both in research and the clinic. In one of the more extensive trials assessing efficacy of pirfenidone in IPF that included patients with short telomeres, pirfenidone was associated with significantly decreased rate of decline in forced vital capacity (FVC) regardless of telomere length status [30]. Similarly, in a recent study of 89 patients with IPF carrying telomere-related gene mutations, treatment with antifibrotics (*n* = 55 pirfenidone, *n* = 34 nintedanib) was associated with a reduced rate of FVC decline. There was no significant difference in efficacy between the two agents [31]. While these and similar studies are encouraging, more extensive studies would be valuable in providing more definitive insight into the benefit of antifibrotics in this population.

Notably, both pirfenidone and nintedanib require regular laboratory monitoring, given the risk of hematologic, hepatic, and other potential toxicities. Side effects are common, often including gastrointestinal symptoms (e.g., diarrhea, particularly with nintedanib) and skin sensitivity (particularly with pirfenidone). Nintedanib should be used cautiously among patients with coronary artery disease and generally avoided if there is active cardiac disease. Further, nintedanib may increase the risk of bleeding and wound healing and may affect international normalized ratio (INR) in patients taking warfarin, so close monitoring is warranted.

Danazol, a synthetic anabolic steroid, is also being actively investigated in patients with TBD-associated ILD. It has been shown to reduce telomere length attrition and is associated with a favorable hematologic response [32]. Small studies have demonstrated that it may also benefit patients with TBD-related lung disease, although small sample sizes and variable treatment responses limit our current understanding. For example, a recently published small prospective trial assessing the efficacy of a danazol equivalent (nandrolone decanoate) included seven patients with baseline ILD. Of these, five demonstrated a positive pulmonary response (*n* = 2 at 12 months, *n* = 3 at 24 months). However, following treatment completion, diffusing capacity remained reduced [33]. Thus, while danazol appears promising, more extensive and more robust studies are necessary to determine whether it should become a standard treatment for these patients. Regular laboratory monitoring is generally recommended due to the risk of hepatotoxicity, and some patients may experience muscle cramps and androgenic effects. Danazol *is contraindicated* in patients with prostatic adenocarcinoma and should be used with caution in patients with benign prostatic hypertrophy, as obstructive symptoms can worsen. Caution is also advised in women with a history of breast cancer, where tumor androgen receptor status should be ascertained before prescribing danazol.

Generally, these medications should be prescribed or overseen by pulmonologists or other subspecialists who have significant experience with their use.

Beyond pharmacotherapies, treatment of TBD-associated ILD should include supplemental oxygen as needed to maintain an oxygen saturation > 88–90%, as with other forms of fibrotic lung disease. Among patients who develop severe ILD and significant hypoxia, evaluation for lung transplantation is frequently warranted. That is discussed elsewhere in this issue.

## Conclusion

ILD is a common and severe manifestation of TBD. The clinical and radiological presentation can be indistinguishable from other forms of ILD and therefore requires a high level of suspicion. Familial history of lung disease as well as features of TBD such as premature graying of the hair, unexplained cytopenias or other hematologic abnormalities, liver cirrhosis, or known telomere conditions such as dyskeratosis congenita are highly suggestive and should prompt further evaluation. Telomere length and gene sequencing of telomere-related genes are crucial to establish the diagnosis. Management and treatment should be individualized and often require a multidisciplinary approach.

## Data Availability

Not applicable.
